# Supplementary material for: Physically asymmetric division of the C. elegans zygote ensures invariably successful embryogenesis
Source: eLife. 2021 Feb 23;10:e61714. doi: 10.7554/eLife.61714 (PMC7972452; doi:10.7554/eLife.61714)
Supplement: Transparent reporting form [file elife-61714-transrepform.docx]

***eLife’s* transparent reporting form**

We encourage authors to provide detailed information *within their submission* to facilitate the interpretation and replication of experiments. Authors can upload supporting documentation to indicate the use of appropriate reporting guidelines for health-related research (see [EQUATOR Network](http://www.equator-network.org/%20)), life science research (see the [BioSharing Information Resource](https://biosharing.org/)), or the [ARRIVE guidelines](http://www.plosbiology.org/article/info:doi/10.1371/journal.pbio.1000412) for reporting work involving animal research. Where applicable, authors should refer to any relevant reporting standards documents in this form.

If you have any questions, please consult our Journal Policies and/or contact us: [editorial@elifesciences.org](mailto:editorial@elifesciences.org).

**Sample-size estimation**

- You should state whether an appropriate sample size was computed when the study was being designed
- You should state the statistical method of sample size computation and any required assumptions
- If no explicit power analysis was used, you should describe how you decided what sample (replicate) size (number) to use

Please outline where this information can be found within the submission (e.g., sections or figure legends), or explain why this information doesn’t apply to your submission:

We aimed for analyzing 10 or more embryos for each condition, which is standard practice in the field. The actual number of embryos analyzed in each case is indicated either directly in figure panels, or else individual data points (embryos) are shown as overlays on top of box plots, where they can be easily counted.

**Replicates**

- You should report how often each experiment was performed
- You should include a definition of biological versus technical replication
- The data obtained should be provided and sufficient information should be provided to indicate the number of independent biological and/or technical replicates
- If you encountered any outliers, you should describe how these were handled
- Criteria for exclusion/inclusion of data should be clearly stated
- High-throughput sequence data should be uploaded before submission, with a private link for reviewers provided (these are available from both GEO and ArrayExpress)

Please outline where this information can be found within the submission (e.g., sections or figure legends), or explain why this information doesn’t apply to your submission:

Individual embryos can be considered as independent biological replicates. Criteria for exclusion/inclusion (e.g. of embryos that were not equalized, i.e. that were not in the 48-53% bracket) are clearly stated in the main text.

**Statistical reporting**

- Statistical analysis methods should be described and justified
- Raw data should be presented in figures whenever informative to do so (typically when N per group is less than 10)
- For each experiment, you should identify the statistical tests used, exact values of N, definitions of center, methods of multiple test correction, and dispersion and precision measures (e.g., mean, median, SD, SEM, confidence intervals; and, for the major substantive results, a measure of effect size (e.g., Pearson's r, Cohen's d)
- Report exact p-values wherever possible alongside the summary statistics and 95% confidence intervals. These should be reported for all key questions and not only when the p-value is less than 0.05.

Please outline where this information can be found within the submission (e.g., sections or figure legends), or explain why this information doesn’t apply to your submission:

The number of embryos in each group, the standard deviations and all p-values are reported in Supplementary Table S6 and Table S2 for lineaged embryos.

To compare sample means for up to 3 groups of embryos, we used Welsh’s independent variance t-test, with Bonferroni-Hochberg correction. For pairwise comparisons of means for more than 3 groups, we used Tukey honest significant difference test, which takes multiple testing into account inherently.

To assess associations between two categorical variables, we used Fisher’s exact test, with results reported in figure legends and N in the figure panels.

Statistical results for correlations are indicated directly in figure panels with p-values and Pearson’s correlation coefficient r, with exception of Figure 3 supplement 1, for which p-values and Pearson’s r are reported in Supplementary Table 3.

All information regarding statistical analysis is mentioned explicitly in the manuscript.

(For large datasets, or papers with a very large number of statistical tests, you may upload a single table file with tests, Ns, etc., with reference to sections in the manuscript.)

**Group allocation**

- Indicate how samples were allocated into experimental groups (in the case of clinical studies, please specify allocation to treatment method); if randomization was used, please also state if restricted randomization was applied
- Indicate if masking was used during group allocation, data collection and/or data analysis

Please outline where this information can be found within the submission (e.g., sections or figure legends), or explain why this information doesn’t apply to your submission:

Groups were formed depending on the genotype (*lin-5(ev571)* vs wild-type), experimental conditions (not upshifted, upshifted at 2-cell stage = control, upshifted during first metaphase to equalize the division), and eventual fate (dead/alive).

For Figures 3-6, we split embryos into either inverted (with AB size < 48%) or equalized (with AB size between 48-53%).

**Additional data files (“source data”)**

- We encourage you to upload relevant additional data files, such as numerical data that are represented as a graph in a figure, or as a summary table
- Where provided, these should be in the most useful format, and they can be uploaded as “Source data” files linked to a main figure or table
- Include model definition files including the full list of parameters used
- Include code used for data analysis (e.g., R, MatLab)
- Avoid stating that data files are “available upon request”

Please indicate the figures or tables for which source data files have been provided:

All data generated and analyzed during this study are included in the manuscript and supporting files.

Results of correlation analysis of relative AB cell size and cell cycle duration in individual cell in lineaged *lin-5(ev571)* embryos are indicated in the Table S3

Table S4 lists p-values for t-test and effect size comparing alive and dead equalized embryos in Figure 4 = supplement 1

Table S7 lists variables used for lasso analysis at different stages in Figure 6, including the model coefficients for best performing model and inclusion frequency,

Source data files and source code files have been provided as individual files attached to the manuscript for Figure 1 – supplements 1-3, Figure 2, Figure 5 and its supplement (the same source data and code files), Figure 6 - figure supplement 1.

The source code and (lineaging) data for remaining figures are available from GitHub <https://github.com/UPGON/worm-rules-eLife> (Figures 3, 4, and 6, and accompanying supplements, as well as Figure 6 - supplement 2 and 3).
